# Basal myokine levels are associated with quality of life and depressed mood in older adults

**DOI:** 10.1111/psyp.13799

**Published:** 2021-03-02

**Authors:** Patrick Mucher, Delgerdalai Batmyagmar, Thomas Perkmann, Manuela Repl, Astrid Radakovics, Elisabeth Ponocny‐Seliger, Ina Lukas, Monika Fritzer‐Szekeres, Johann Lehrner, Thomas Knogler, Dimiter Tscholakoff, Martina Fondi, Oswald F Wagner, Robert Winker, Helmuth Haslacher

**Affiliations:** ^1^ Department of Laboratory Medicine Medical University of Vienna Vienna Austria; ^2^ FH Campus Wien University of Applied Sciences Vienna Austria; ^3^ Center of Public Health Medical University of Vienna Vienna Austria; ^4^ Empirical Research Vienna Austria; ^5^ Health and Prevention Center Sanatorium Hera Vienna Austria; ^6^ Department of Neurology Medical University of Vienna Vienna Austria; ^7^ Institute for Imaging Diagnostics Sanatorium Hera Vienna Austria; ^8^ Radiologie Hernals Vienna Austria

**Keywords:** athletes, late‐life depression, MRI, myokines, physical activity

## Abstract

In an aging society, late‐life depression has become an increasing problem. There is evidence that physical activity ameliorates depressive symptoms and increases the quality of life (QoL). However, the underlying mechanisms are still poorly understood. Myokines are molecules secreted in response to muscle contraction. Some of them can cross the blood‐brain barrier, making them promising candidates for mediating the beneficial effects of physical activity on mood. The present study aims to compare circulating myokine levels to depression/QoL in older athletes and controls. 55 athletes, 57 controls >59 years were enrolled. The assessment included ergometry, magnetic resonance imaging, blood withdrawal, and neuropsychological testing. Serum interleukin‐6 (IL‐6), irisin, brain‐derived neurotrophic factor (BDNF), kynurenine, and cathepsin B were analyzed and compared to surrogates of depression and quality of life. Athletes presented with higher levels of Cathepsin B. Among controls, all myokines but irisin were associated with age. Also, among controls, kynurenine and IL‐6 correlated inversely with specific dimensions of quality of life questionnaires, and IL‐6 further with depressive symptoms and decreased physical performance. No such associations could be found among athletes. Irisin levels were inversely associated with mild depression and low‐grade white matter‐lesions in the brain and predicted impaired QoL. The circulating levels of several myokines/muscle activity‐related factors appear to be associated with depressive symptoms and impaired QoL among older adults. However, in athletes, some of these connections seem ameliorated, suggesting additional stressors (as f.e. age) or a different pathomechanism among athletes.

## Impact Statement

Physical exercise positively affects mood and quality of life, however, the underlying mechanisms are not yet fully understood. Myokines are released in response to muscle contraction. They might be promising candidates for mediating muscle‐brain‐crosstalk. Our results suggest that basal kynurenine and interleukin‐6 levels are correlated with poor quality of life and/or depressive symptoms. In contrast, low irisin levels were associated with mild depression and low‐grade white matter‐hyperintensities in brain MRIs and they predicted impaired quality of life.

## 1 INTRODUCTION

It is well established that continual physical activity goes along with a better quality of life (Batmyagmar et al., 2019; Boldt et al., 2018; Sillanpää et al., 2012) and less depressive symptoms (Archer et al., 2014; Haslacher et al., 2015a). This effect is mainly seen in older adults (Sjösten & Kivelä, 2006), who might suffer from depression or report poor quality of life often due to various comorbidities. The mechanisms by which muscle activity influences brain physiology are still poorly understood. During the last years, several compounds termed “myokines” were identified that might mediate the beneficial effects of physical activity (Pedersen, 2019). During physical activity, these myokines and muscle‐activity‐related factors are secreted into the blood circulation and eventually cross the blood‐brain‐barrier. In return, this activity‐related secretion acts on their basal levels (Janikowska et al., 2020; Moon et al., 2016). Therefore, it could be hypothesized that circulating levels of those compounds might serve as a surrogate for depressive symptoms and quality of life.

As stated above, physical activity has been shown to positively affect depressive symptoms and reduced quality of life. For instance, sedentary older adult report significantly higher levels of anxiety and depression and reduced quality of life when compared to physically active individuals (de Oliveira et al., 2019). This holds for various intensities of physical activity, as, for example, Yoga interventions (Noradechanunt et al., 2017), flexibility exercise (Byeon, 2019) or marathon sports (Batmyagmar et al., 2019). There are several hypotheses regarding the pathogenesis of depression, including the monoamine hypothesis, the vascular depression hypothesis, and the cytokine hypothesis (Khalaf et al., 2015; Marathe et al., 2018). Especially late‐life depression is often accompanied by structural changes in the central nervous system, as, for example, by gliosis, which presents as white matter lesions in MRI, which most likely result from cerebrovascular disease and increased inflammation (Khalaf et al., 2015). Especially the cytokine and the vascular hypotheses offer a target for circulating messenger and effector molecules, whose concentration and/or composition could be mediated by physical activity.

In this regard, previous mechanistic research has identified several molecular that are affected by muscle activity. Some of them are regulated by PGC1α, whose expression is induced by physical activity (Pedersen, 2019). In murine muscles, overexpression of PGC1α enhances the expression of fibronectin type III domain‐containing protein 5 (FNDC5). FNDC5 then increases plasma irisin levels, which might overcome the blood‐brain barrier, stimulating BDNF (Brain‐derived neurotrophic factor) expression within the central nervous system (Bostrom et al., 2012). Indeed, an association between physical activity and circulating irisin levels was also reported for humans (Wrann, 2015). Above that, PGC1α upregulates kynurenine aminotransferase, an enzyme that produces kynurenic acid from kynurenine (KYN) generated in the tryptophan metabolism (Muller & Schwarz, 2007; Schwarcz et al., 2012). KYN is a neurotoxin that induces neuronal apoptosis and inflammation. Hence, dysregulations of the KYN metabolism are associated with depression (Claes et al., 2011; Myint & Kim, 2014). The conversion to kynurenic acid prevents the molecule from crossing the blood‐brain barrier (Schwarcz et al., 2012). Moreover, PGC1α levels are inversely correlated with Interleukin‐6 (IL‐6) concentrations (Handschin et al., 2007).

Although it is better known as a cytokine, IL‐6 was one of the first molecules defined as a myokine (Pedersen & Febbraio, 2008). IL‐6 is not only produced by macrophages but also expressed in myoblasts, and it can be secreted by muscle cells without activating the pro‐inflammatory pathway (Bartoccioni et al., 1994; De Rossi et al., 2000). Whether the cytokine develops a pro‐ or anti‐inflammatory character mainly depends on the environment and whether IL‐6 is expressed acutely or chronically (Pedersen & Febbraio, 2008). Hence, acute IL‐6 expression seen in athletes goes along with low basal levels of inflammatory cytokines during resting periods (Janikowska et al., 2020). As the inflammation hypothesis of late‐life depression suggests, an imbalance between pro‐ and anti‐inflammatory signals impairs neurotoxins' clearance and reduces neuron density (Alexopoulos, 2019). Indeed, patients with major depression exhibit higher circulating IL‐6 levels than healthy controls, and IL‐6 levels decrease in response to treatment (Goldsmith et al., 2016; Jin et al., 2020).

Physical activity‐induced upregulation of PGC1α is also accompanied by a rise in Cathepsin B (CTSB) levels (Karlsson et al., 2019), although a causal relationship between those molecules has not yet been established. CTSB is a protein belonging to the family of lysosomal cysteine proteases, which can be detected at high levels in various types of human cancer (Aggarwal & Sloane, 2014). However, it could be shown for mice, rhesus monkeys, and humans that CTSB is increased by physical activity. CTSB induces BDNF expression, and it is not surprising that CTSB concentrations were associated with better fitness and hippocampus‐dependent memory function (Moon et al., 2016).

Together, there is increasing evidence that physical activity's beneficial effect on depressive symptoms and quality of life might be at least partially attributable to so‐called myokine action. For this, myokines and muscle‐activity‐related factors might be distributed by the bloodstream and pass the blood‐brain‐barrier. As a consequence, the peripheral concentrations of these molecules might be associated, whether causal or not, with depressive symptoms and reduced quality of life. The effect sizes, however, might be low to medium, as most of these mediators are not produced by the contracting muscle alone, but by various cells and tissues and their function varies with their place of action. Irisin has been found in non‐small cell lung cancer cells and stromal fibroblasts (Nowinska et al., 2019), Kynurenine aminotransferases, for instance, are expressed in the central nervous system as well (Song et al., 2018), however, intrathecally produced kynurenic acid is unlikely to affect circulating kynurenine levels. Kynurenine itself can be produced by the skin as well (Sheipouri et al., 2012). CTSB is not only produced in response to muscle activity, but for instance also by macrophages during perineural invasion (Bakst et al., 2017), cartilage cells (Zwicky et al., 2002), by various types of cancer, where high CTSB expression often goes along with a less favorable prognosis (Chan et al., 2010; Ozeki et al., 1993; Ruan et al., 2016), and by microglia in the central nervous system (Ni et al., 2019). Regarding IL‐6, for example, it might not be expected that athletes feature higher basal levels. In contrast, it can be hypothesized that microinflammation during the marathon might induce anti‐inflammatory pathways as a counter‐regulatory response, by which basal circulating IL‐6 levels are kept low (Janikowska et al., 2020), which is also supported by the short half‐life of the cytokine. A relevant part of peripherally measurable BDNF origins from platelets (Hochstrasser et al., 2013; Türck & Frizzo, 2015), which impairs the interpretation of serum BDNF levels. These could rather reflect a BDNF secretion capacity than the values actually present in the central nervous system. Howeover, molecules acting as myokines might not only be secreted by tissues other than muscle, but also dependend on physiological states. One potential confounder is age, as the peripheral levels of several myokines change with advanced age (de Bie et al., 2016; Ferrucci et al., 2005; Refaey et al., 2017; Ruan et al., 2019; Wei et al., 1992; Wyczałkowska‐Tomasik & Pączek, 2012). Moreover, the amount of adipose tissue might influence the levels of cytokines (Carey et al., 2004), and must, therefore, be considered when interpreting the results.

As stated above, at least part of the circulating levels might be attributable to muscle activity, and it cannot be excluded that circulating levels might serve as surrogate markers for muscle‐brain interaction. The present study, therefore, aims to investigate whether basal concentrations of myokines or muscle‐activity‐related factors (IL‐6, CTSB, KYN, irisin, and BDNF) i) are differently associated with lifestyle‐ and physiological characteristics among athletes and controls, and if in either of the groups these circulating myokine levels are useful for predicting ii) reduced quality of life or iii) depressive symptoms, and associated features in imaging data of the central nervous system, respectively.

## 2 METHOD

### 2.1 Study design and participants

This study follows a retrospective, exploratory cross‐sectional design, and reverts to the cohorts of the Vienna Marathon Trial (Batmyagmar et al., 2019; Winker et al., 2010), which were prospectively enrolled in 2009. Back then, 63 older marathon athletes and 73 control participants, who did not differ in terms of sex, age, and education, were screened. Of those, 56 athletes and 58 controls met all inclusion criteria (inclusion criteria: [a] participation in one of the three listed marathons during the preceding two years, [b] weekly amount of training ≥ 2 hr, [c] at least in the 60th year of life [age ≥ 59]; exclusion criteria: [a] present or past exposure to neurotoxic substances, [b] not German as a native language, [c] diseases that markedly affect CNS functions: cerebrovascular stroke, brain tumor, depression, Alzheimer's disease, epilepsy, multiple sclerosis, Parkinson's disease, etc., [d] manifest cardiovascular disease, [e] chronic alcoholism, [f] unwillingness to give informed consent). Of 55 athletes and 57 controls, biomaterial was available to quantify myokines and muscle‐activity‐related factors (IL‐6:1 missing data point because of insufficient material). Those were included in the present analysis.

In brief, the examinations started between 10:00 and 10:30 a.m. to minimize circadian variability. After the recording of biographic and biometric data and medical history, participants underwent a medical check‐up performed by a specialist in internal medicine. Subsequently, blood was drawn for routine analyses and part of which was sent to the MedUni Wien Biobank, as described below. Then, physical performance was assessed by ergometry. After this, participants were asked to complete neuropsychological test batteries and questionnaires (Winker et al., 2010).

### 2.2 Neuropsychological assessment and imaging

Depressive symptoms were assessed by the Beck Depression Inventory (Beck et al., 1961) and the Geriatric Depression Scale (Alexopoulos et al., 1993). Reductions in Quality of Life (QoL) perception were screened using the WHO‐5 Well‐being‐index (Heun et al., 2001). Domain‐specific impairments in different QoL dimensions were queried using the SF‐36 clinical questionnaire (Larson, 1997). Lifestyle specifics were assessed using the Personal Lifestyle Questionnaire (PLQ) with 24 items (Brown et al., 1983). Each of the items had to be rated from 0 (never) to 3 (nearly always), or indicated whether it was not applicable (e.g., monthly breast examination). An experienced clinical psychologist evaluated all tests.

Magnetic resonance imaging was performed on a Siemens Symphony 1.5 T (Siemens, Erlangen, Germany) using a standard head coil (29). The protocol included the following: (a) axial FLAIR (fluid‐attenuated inversion recovery): TR 696 msec, TE 24 msec, 5 mm slice thickness, distance factor 20%, FOV (field of view) 183 × 230, number of slices 20, resolution 256 × 224. (b) axial T2* flash 2d: TR 477, TE 12 msec, 5 mm slice thickness, distance factor 20%, FOV 183 × 210, number of slices 20, resolution 448 × 512. (c) axial T1 TSE (turbo spin‐echo sequence) TR 477 msec, TE 12 msec, 5 mm slice thickness, distance factor 20%, FOV 196 × 210, number of slices 20, resolution 228 × 256. (d) coronal T2 TSE: TR 4,480 msec, TE 94 msec, high resolution (perpendicular to the hippocampus), 2 mm slice thickness, distance factor 20%, FOV 186 × 230, number of slices 24, resolution 198 × 256. (e) coronal 3D MPRAGE: TR 1,420 msec, TE 3.2 msec, slice thickness (partition) 3 mm, FOV 178 × 260 number of slices 36, resolution 316 × 512. The images were used in conjunction with a board‐certified radiologist to manually rate the white matter lesions as “not present,” “isolated lesions,” or “pronounced changes.”

### 2.3 Physical performance test

Ergometry was supervised by trained medical personnel. Individual working capacity was calculated as a percentage of the predicted (=100% workload) Watt value (derived from the tabulation, standardized for sex, age, and body surface (Böhm et al., 1978)). Briefly, the workload was increased every two minutes in steps of 25 W, beginning with 25 W and going on until the point of exhaustion on an Ergometrics 900 (Ergoline GmbH, Bitz, Germany). The individual physical working capacity (PWC) was expressed as the individual maximal power (Watt_max_) in percent of a reference value (Watt_ref_): PWC_ind_ = 100 × Watt_max_/Watt_ref_ (Böhm et al., 1978).

### 2.4 Laboratory analyses

At the time of inclusion, blood was drawn and submitted to the MedUni Wien Biobank, a central facility at the Medical University of Vienna specialized in the processing and storage of human biomaterial (Haslacher et al., 2018). There, blood serum was prepared and stored at median temperatures <−70°C until analysis.

BDNF was quantified in 2010 from frozen sera through enzyme‐linked immunosorbent assays (ELISA) purchased from Ray Biotech Inc. (Norcross, USA) as described earlier (Winker et al., 2010). All other parameters were measured in 2020 from banked sera. IL‐6 was quantified with Roche Elecsys® IL‐6 electrochemiluminescence on a Cobas e602 analyzer immunoassays (Roche, Rotkreuz, Switzerland) at the Department of Laboratory Medicine, Medical University of Vienna, in a certified (ISO 9001:2015) and accredited (ISO 15189:2012) environment. KYN was quantified using a commercially available, CE/IVD‐marked competitive ELISA kit (IDK® Kynurenine K7728, Immundiagnostik, Bensheim, Germany). ELISAs measured CTSB (Human Cathepsin B ELISA kit ab119584, Abcam, Cambridge, UK) and irisin (competitive Irisin ELISA RAG018R, BioVendor, Brno, Czech Republic). ELISAs were performed in single determinations after the tests’ intra‐assay variability was verified in duplicates. Due to a considerable between‐assay variability for CTSB and irisin, which hampered the comparison of results derived from different ELISA plates, measurement results were z‐standardized assay‐wise, and only z‐standardized values were compared. To ensure comparability, both athletes and control samples were applied on each assay plate, and overall results were interpreted together with assay‐wise results. This approach was not necessary for CE‐marked tests (kynurenine, IL‐6) and BDNF, as for the latter already available data from previous analyses were used.

### 2.5 Statistical analyses

Continuous data are presented as median (interquartile range) and categorical data as counts (percentages). As stated above, z‐standardized values of CTSB and irisin were calculated assay‐wise and were included in the calculations instead of the resulting concentrations. Mann‐Whitney U tests compared differences in myokine levels and other continuous data between athletes and controls. Differences in myokine levels between two dichotomous factors (e.g., group and BDNF category) were assessed by 2 × 2 ANOVA. Since variables did not meet the normality assumption required for analyses of variances, ANOVA was performed on ranks instead of actual numbers as suggested by Brownie and Boos (1994). Pearson's *χ* ^2^ tests assessed differences in categorical variables. Predictive values were evaluated by binary logistic regression models (odds ratios are given ± 95% confidence intervals [95%CI]) and areas under the receiver‐operating‐characteristic (ROC)‐curves (AUC, given ± 95%CI) were interpreted. All calculations were performed using MedCalc v19.4.1 (MedCalc Software Ltd, Ostend, Belgium), graphs were drawn with GraphPad Prism 8.4.2 (GraphPad, La Jolla, USA). *p* values <.05 were considered statistically significant. Due to the exploratory nature of the study, no correction of *p* values for multiple testing was performed.

## 3 RESULTS

### 3.1 Cohort characteristics

Baseline characteristics of 55 athletes and 57 control participants are listed in Table 1. As intended, controls and athletes did not differ in age, sex, and education years. Athletes presented with considerably higher physical performance and a lower BMI. In terms of psychological test systems, athletes yielded more favorable scores in both BDI and GDS and the WHO‐5 questionnaire and all SF‐36 dimensions except for emotional well‐being. When comparing lifestyle habits between athletes and controls, it turned out that both groups differ in terms of physical activity and diet (items “Personal fitness program,” “Climb 5 stairs or walk 1.5 km /day,” “3 times sports per week,” and “Maintain weight within desirable limits,” “Balanced nutrition,” “Adding salt to prepared food”). Beyond these expected differences, athletes reported more time for physical intimacy (*p* = .014) and had a slightly stricter attitude toward not to drive after drinking alcohol (*p* = .049). However, there were no other significant differences concerning health promotion, relaxation, safety, and substance use.

**TABLE 1:** Baseline characteristics of athletes and controls

	Athletes (*n* = 55)	Controls (*n* = 57)	*p* value
*Biometry and physical performance*			
Age, years	66 [62–68]	66 [63–69]	*U* = 1,510.0; *p* = .737
Female Sex [%]	5 (9%)	6 (11%)	*χ* ^2^ = 0.065; *p* = .800
Education, years	9 [8–13]	10 [8–16]	*U* = 1,441.0; *p* = .444
Training intensity [hr/week]	7.0 [95%CI 5.8–8.2]		
Year of first marathon (examination in 2009)	1991 (1985–1998)		
Year of best marathon	1999 (1992–2003)		
Best completion time			
Marathon (*N* = 45)	3:30 (3:12–3:55)		
Half‐marathon (*N* = 4)	Range: 1:30–2:03		
Bicycle marathon (*N* = 5)	Range: 1:00–1:55^a^		
Triathlon (*N* = 1)	Not specified		
Ergometer performance [W]	**200 [175–238]↑**	**150 [123–175]↓**	***U* = 446.5; *p* < .0001**
Ergometer performance [%]	**152 [128–169]↑**	**99 [85–115]↓**	***U* = 245.5; *p* < .0001**
BMI, kg/m^2^	**23.3 [22.4–25.0]↓**	**26.2 [24.6–29.3]↑**	***U* = 651.0; *p* < .0001**
*Neuropsychological diagnostics*			
BDI	**3 [1–7]↓**	**7 [5–10]↑**	***U* = 896.5; *p* < .0001**
GDS	**0 [0–1]↓**	**1 [0–3]↑**	***U* = 1,191.0; *p* = .017**
WHO‐5 Well‐being index	**20 [18–22]↑**	**19 [16–20]↓**	***U* = 1,039.0; *p* = .005**
SF‐36 General Health Perception	**82 [72–95]↑**	**72 [58–87]↓**	***U* = 1,091.5; *p* = .005**
SF‐36 Physical functioning	**100 [95–100]↑**	**90 [80–96]↓**	***U* = 700.0, *p* < .0001**
SF‐36 Physical role function	**100 [100–100]↑**	**100 [50–100]↓**	***U* = 1,114.5, *p* = .0002**
SF‐36 Bodily pain	**100 [84–100]↑**	**84 [62–100]↓**	***U* = 1,098.5, *p* = .004**
SF‐36 Vitality	**80 [73–90]↑**	**70 [60–80]↓**	***U* = 930.5, *p* = .0003**
SF‐36 Emotional well‐being	84 [80–88]	80 [72–89]	*U* = 1,298.0, *p* = .153
SF‐36 Emotional role functioning	**100 [100–100]↑**	**100 [83–100]↓**	***U* = 1,253.0, *p* = .008**
SF‐36 Social functioning	**100 [100–100]↑**	**100 [75–100]↓**	***U* = 1,128.0, *p* = .003**
*Lifestyle (PLQ), 4‐point‐rating from never (0) to nearly always (3)*			
Annual medical examination	3 (2–3)	3 (2–3)	*U* = 1566.5, *p* = .994
Meeting with friends	2 (2–2)	2 (2–3)	*U* = 1524.5, *p* = .920
Regular meals	3 (2–3)	3 (2–3)	*U* = 1566.5, *p* = .994
Uses security belt in car	3 (3–3)	3 (3–3)	*U* = 1,459.5, *p* = .159
Balanced nutrition	**3 (2–3)↑**	**3 (2–3)↓**	***U* = 1,214.0, *p* = .027**
Conversations about personal matters	2 (1–3)	2 (1–2¼)	*U* = 1,436.0, *p* = .885
Drink and drive	**0 (0–0)↓**	**0 (0–1)↑**	***U* = 1,297.5, *p* = .049**
Emergency phone numbers	2 (1–3)	2 (1–3)	*U* = 1,414.0, *p* = .653
Sufficient sleep	3 (2–3)	3 (2–3)	*U* = 1,479.5, *p* = .684
Personal fitness program	**3 (3–3)↑**	**1 (1–2)↓**	***U* = 428.0, *p* < .0001**
Climb 5 stairs or walk 1.5 km/day	**3 (3–3)↑**	**2 (1–3)↓**	***U* = 834.0, *p* < .0001**
Adhere to speed limit when driving	3 (2–3)	3 (2–3)	*U* = 1,373.0, *p* = .434
Daily consumption of cigarettes	0 (0–0)	0 (0–0)	*U* = 1,428.5, *p* = .054
Adding salt to prepared food	**0 (0–1)↓**	**1 (0–1)↑**	***U* = 1,157.7, *p* = .008**
Daily relaxing (15–20 min)	2 (2–3)	2 (2–3)	*U* = 1,448.5, *p* = .459
Daily alcohol consumption	1 (0–1)	1 (0–1)	*U* = 1,480.0, *p* = .574
3 times sports per week	**3 (3–3)↑**	**1 (1–2½)↓**	***U* = 429.0, *p* < .0001**
Time for physical intimacy	**2 (1½–3)↑**	**2 (1–2)↓**	***U* = 1,119.5, *p* = .014**
Limit caffeine intake to 3 cups/day	1 (0–3)	1 (½–3)	*U* = 1503.5, *p* = .828
Smoking in bed	0 (0–0)	0 (0–0)	*U* = 1,485.0, *p* = .317
Annual dental checkup	3 (3–3)	3 (2–3)	*U* = 1,427.5, *p* = .283
Monthly breast examination	1 (1–3), *N* = 5	1½ (¾–2), *N* = 6	*U* = 12.0, *p* = .561
Maintain weight within desirable limits	**3 (3–3)↑**	**2 (2–3)↓**	***U* = 920.5, *p* < .0001**
Avoiding alcohol when taking medication	3 (3–3)	3 (2–3)	*U* = 1,405.5, *p* = .273

Note

For more than 5 data points, continuous data are given as medians (interquartile ranges) and compared by Mann‐Whitney tests. For significant results, arrows indicate whether the respective rank sum was higher (↑) or lower (↓) than that of the opposite group. Categorical data are presented as counts (percentages) and compared by Pearson's *χ* ^2^ tests.

Bold values indicate significant differences between the values given in columns two and three: median (interquartile range) or counts (percentage). Column four: test statistics (Mann‐Whitney‐U) and p‐value.

Abbreviation: PLQ, Personal Lifestyle Questionnaire.

^a^ 3 data points missing.

### 3.2 Association of basal myokines levels with biometric data in athletes and controls

Basal levels were measured in blood samples taken late in the morning and stored at <−70°C until analysis. As stated above, levels of CTSB and irisin were z‐standardized assay‐wise to reduce inter‐assay variability. A comparison between athletes and controls is presented in Table 2. In brief, basal levels of IL‐6, KYN, BDNF, or irisin (z‐standardized) did not differ between athletes and controls, whereas z‐standardized CTSB was significantly higher in athletes (Hodges‐Lehmann median difference of z values = 0.6 [95% CI: 0.3–0.9], *p* = .0004).

**TABLE 2:** Basal myokine/muscle‐activity‐related factors in athletes and controls

Basal myokine levels	Athletes	Controls	Difference
IL‐6 [pg/ml]	0.75 [0.75–2.46]	1.54 [1.75–2.78]	*U* = 1,477.0; *p* = .694
Kynurenine [µmol/L]	2.8 [2.4–3.2]	2.8 [2.3–3.9]	*U* = 1564.5; *p* = .986
BDNF [ng/ml]	16.9 [12.4–24.8]	16.0 [8.4–23.6]	*U* = 1,409.5; *p* = .358
Cathepsin [B *z*‐score]	**0.11 [−0.29–1.02]**	**−0.46 [−0.87–0.06]**	***U* = 962.5; *p* < .001**
Irisin [*z*‐score]	−0.13 [−0.86–0.33]	−0.02 [−0.63–0.65]	*U* = 1,359.0; *p* = .225

Note

Continuous data are given as medians (interquartile ranges) and compared by Mann‐Whitney tests.

Bold values indicate significant differences between the values given in columns two and three: median (interquartile range) or counts (percentage). Column four: test statistics (Mann‐Whitney‐U) and p‐value.

Among athletes, CTSB levels were negatively associated with BMI (*ρ* = −0.397, *p* = .003). However, controlling for BMI in an ANOVA on CTSB ranks did not affect the fact that CTSB was higher in athletes than in controls (mean rank difference: 16.6, *p* = .011).

Several correlations between myokines or muscle activity‐induced factors became apparent within groups (athletes/controls). In this regard, all myokines/muscle activity‐induced factors but BDNF were rising with age (Table 3). Moreover, higher IL‐6 levels were associated with significantly worse absolute physical performance (*ρ* = −0.432, *p* = .008) and higher BMI (*ρ* = 0.435, *p* = .001), again only in controls (athletes: *p* = .167, *p* = .224). In contrast, there was in athletes a trend for an inverse correlation between KYN and BDNF (*ρ* = −0.254, *p* = .062), which was in‐turn trend‐wise positively associated with training intensity [hr/week] (*ρ* = 0.263, *p* = .053). However, there was no such correlation among controls.

**TABLE 3:** Basal myokine/muscle‐activity‐related factors and their correlation with age

Correlation with age	Athletes	Controls	Difference in *ρ*
IL‐6 [pg/ml]	*ρ* = 0.181, *p* = .189	***ρ* = 0.380, *p* = .004**	*Z* = −1.11, *p* = .266
Kynurenine [µmol/L]	*ρ* = 0.197, *p* = .150	***ρ* = 0.362, *p* = .006**	Z = −0.94, *p* = .355
BDNF [ng/ml]	*ρ* = 0.039, *p* = .776	*ρ* = −0.105, *p* = .439	*Z* = 0.743, *p* = .457
Cathepsin [B *z*‐score]	*ρ* = −0.227, *p* = .096	***ρ* = 0.327, *p* = .013**	***Z* = −2.94, *p* = .003**
Irisin [*z*‐score]	*ρ* = −0.109, *p* = .429	***ρ* = 0.331, *p* = .012**	***Z* = −2.33, *p* = .020**

Note

The column “Difference in *ρ*” indicates whether Spearman's *ρ* are significantly different between groups.

Bold values indicate statistically significant Spearman’s *ρ*.

Hence, it appears as if in athletes, levels of most assessed myokines and muscle‐activity‐related factors might be uncoupled from other physiological characteristics, like f.e. age, and physical performance. In contrast, there was a weak and statistically non‐significant relationship between the weekly amount of training and serum BDNF levels and a non‐significant inverse association between serum BDNF and the neurotoxin KYN that could be seen only in athletes, which moreover presented with comparatively higher levels of CTSB. In the next steps, we aimed to assess whether basal myokine levels are also related to depressive symptoms and quality of life in either group.

### 3.3 Basal myokine levels and quality of life in athletes and controls

**FIGURE 1:**
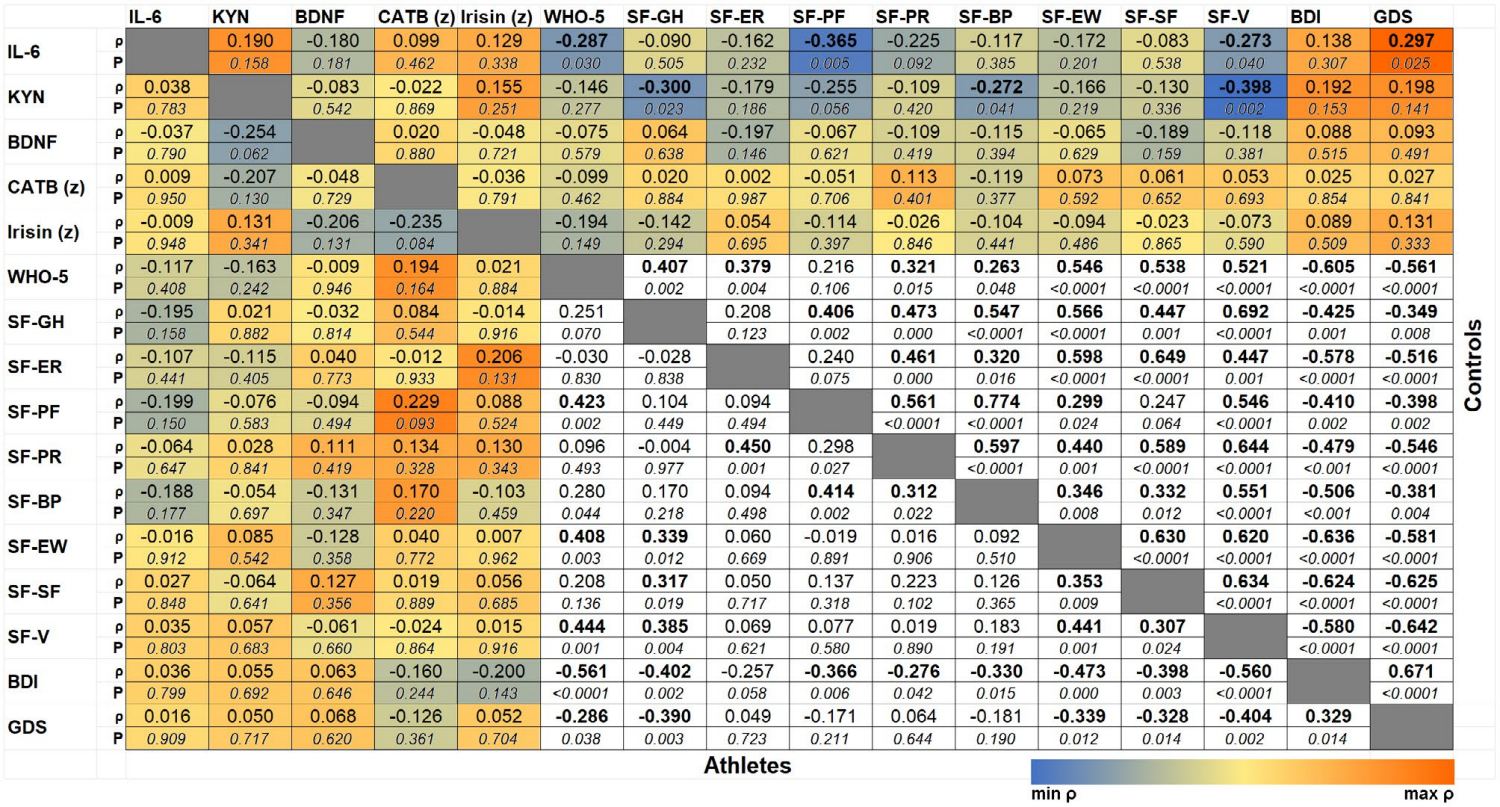
Correlogram of Spearman's rank correlations between myokines/muscle‐activity‐related factors and Quality of Life (WHO‐5 Well‐being index; SF‐36 domains General health perception, GH; Emotional role functioning, ER; Physical functioning, PF; Physical role functioning, PR; Bodily pain, BP; Emotional well‐being, EW; Social functioning, SF; Vitality, V) or Depression scales (Beck Depression Inventory, BDI; Geriatric Depression Scale, GDS). The correlogram is divided by a grey border: the bottom‐left‐sided values were derived from athletes, the top‐right‐sided values from control individuals. For correlations including myokine levels, cell colors indicate the extent and the direction of the correlation (with the minimum observed *ρ* in blue and the maximum observed *ρ* in red). *p* values are presented below the Spearman's *ρ* in italic letters, correlation coefficients with *p* <.05 are highlighted by bold letters

Among controls, significant inverse correlations between KYN levels and the subscales general health perception (*ρ* = −0.300, *p* = .023), bodily pain (*ρ* = −0.272, *p* = .041), vitality (*ρ* = −0.398, *p* = .002), as well as a trend‐wise correlation with the subscale physical functioning (*ρ* = −0.255, *p* = .056) of the SF‐36 appeared. Physical functioning (*ρ* = −0.365, *p* = .005) and vitality (*ρ* = −0.273, *p* = .040) correlated with basal IL‐6 levels as well. Moreover, basal IL‐6 levels were negatively associated with the WHO‐5 questionnaire (*ρ* = −0.287, *p* = .030). A correlogram is shown in Figure 1.

When compiling a binary logistic regression model for the appearance of suspicious WHO‐5 Well‐being scores ≤ 12.5 (50%), irisin turned out as a significant predictor (*p* = .018). Results from regression analyses are summarized in Table 4.

**TABLE 4:** Prediction of suspicious BDI and WHO‐5 scores by group status (athlete/control) and myokine concentrations

Model summary	Prediction of BDI ≥ 10		Prediction of WHO−5 ≤ 50%	
	Statistics	*p* value	Statistics	*p* value
Omnibus test	***χ*^2^ = 14.163, *df* = 6**	**.029**	***χ*^2^ = 14.413, *df* = 6**	**.025**
Nagelkerke's *R* ^2^	0.187		0.303	
Nagelkerke's *R* ^2^ without non‐significant predictors	0.087		0.175	

Predictors	Coefficient B	*p* value	Coefficient B	*p* value
IL−6 [pg/ml]	0.035 ± 0.032	.280	0.069 ± 0.041	.091
Kynurenine [µmol/L]	0.245 ± 0.143	.088	0.077 ± 0.198	.696
BDNF [ng/ml]	0.036 ± 0.027	.188	0.040 ± 0.044	.363
Cathepsin [B *z*‐score]	−0.049 ± 0.271	.857	−0.160 ± 0.497	.747
Irisin [*z*‐score]	−0.449 ± 0.286	.117	**0.862 ± 0.364**	**.018**
Group (athletes/controls)	**−1.339 ± 0.573**	**.019**	−1.773 ± 1.234	.151
*Constant*	−2.405 ± 0.783	.002	−3.723 ± 1.302	.004

Note

Nagelkerke's *R^2^* is given for the model including all predictors, as well as for a model including only the significant predictor.

Bold values indicate statistically significant omnibus tests or regression coefficients.

Basal myokine levels, depressive symptoms, and associated MRI features in athletes and controls.

**FIGURE 2:**
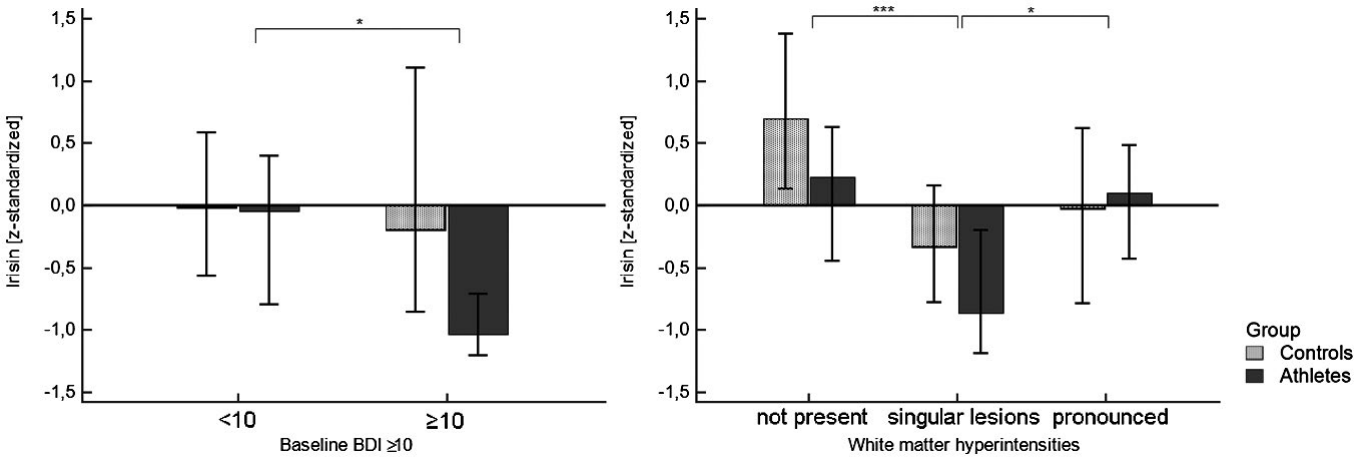
Comparison of z‐standardized irisin levels between individuals with BDI (Beck's Depression Inventory) scores <10 and ≥10 (a) and between individuals with no, low‐grade or high‐grade white matter lesions in brain MRI. Data were compared by Two‐way analyses of variance (Two‐way‐ANOVA) on ranks of irisin. Test results for a main effect of the x‐axis category: *p <.05, ***p <.001

IL‐6 levels were associated with quantitative scores of the Geriatric Depression Scale (*ρ* = 0.297, *p* = .025) in controls. Among athletes, no such correlation could be found. When comparing irisin levels between athletes and controls with BDI scores above or below 10, which are considered suspicious, BDI scores ≥ 10 (*F* = 4.050, *df*_1_ = 1, *df*_2_ = 108, *p* = .047) and whether individuals were athletes or controls (*F* = 5.101, *p* = .026) presented with a significant main effect regarding circulating irisin concentrations (Figure 2).

**FIGURE 3:**
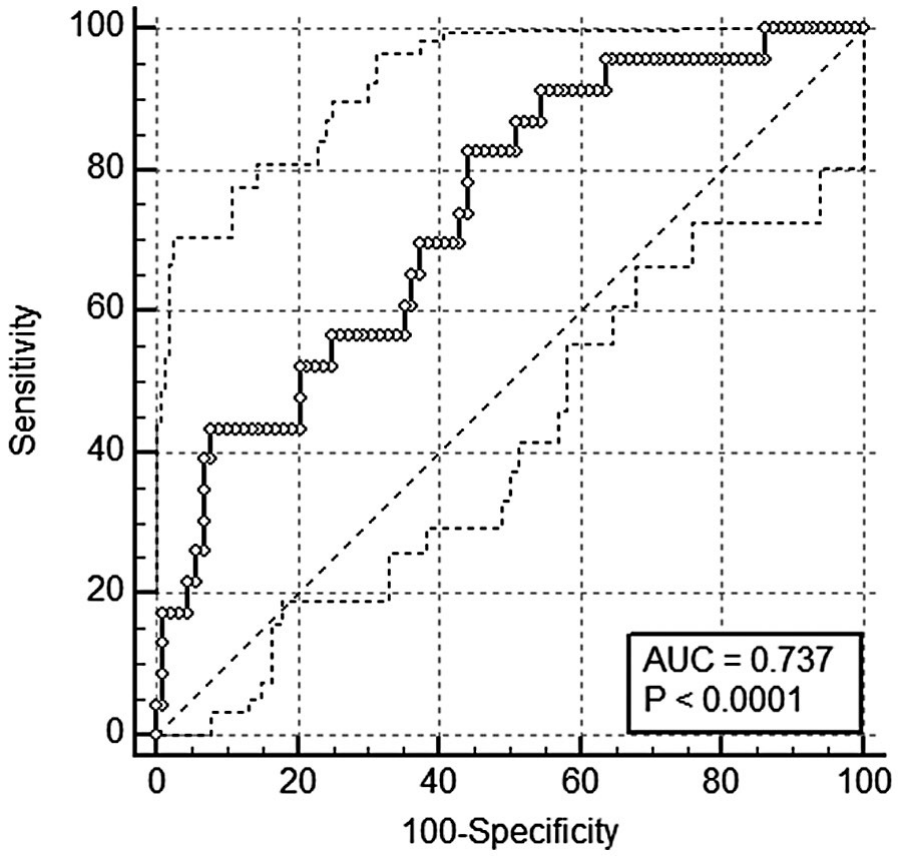
ROC‐curve for a binary logistic regression model, including group assignment (athletes/controls), standardized irisin, and cathepsin B and interleukin‐6‐, kynurenine‐ and BDNF levels

A binary logistic regression model providing group assignment (athletes/controls), z‐standardized irisin and CTSB, as well as IL‐6‐, KYN‐ and BDNF levels, yielded statistical significance (*χ* ^2^ = 14.165, *df* = 6, *p* = .028). Within the model, only group assignment presented as a significant predictor.

(odds ratio 0.262 [95% CI: 0.085–0.806], *p* = .019 for athletes). However, the resulting predictive capability of the model including the myokines (ROC‐AUC = 0.737 ± 0.057, *p* <.0001, Figure 3) was significantly greater than that derived from predicting suspicious BDI scores by group assignment alone (difference between areas = 0.095 ± 0.044, *p* = .033).

Regarding associations with structural changes within the central nervous system, irisin levels were significantly predicted by the presence of white matter lesions in MRI (*F* = 10.438, *df*_1_ = 2, *df*_2_ = 105, *p* <.001). In detail, irisin levels were significantly lower in individuals with isolated gliosis compared to both individuals with no white matter lesions (*p* <.001) and pronounced gliosis (*p* = .010), see Figure 2. The inclusion of age, BMI or HbA_1c_, as a surrogate of insulin resistance, as covariates did not significantly affect this relationship (*p* = .302, *p* = .236).

## 4 DISCUSSION

Physical activity is associated with an improved quality of life and fewer depressive symptoms. For this reason, exercise programs are among the accepted therapeutic approaches today, also for older adults. There is evidence that crosstalk between the skeletal muscle and the brain mediates at least part of physical activity's positive effect on mood and mental health. The present study confirmed that basal levels of some molecules that are either themselves considered "myokines" or are modified by myokines, namely KYN, IL‐6, and irisin, are associated with impairments of quality of life or depressed mood. However, this association appeared to be disrupted in athletes, suggesting a more complex regulatory mechanism in athletes that affects basal circulating myokine levels.

### 4.1 Irisin predicts surrogates of depression and reduced quality of life

Low z‐standardized irisin levels were associated with suspicious BDI scores (≥10) at baseline (preferably in athletes than in controls), and presented as significant predictors of reduced well‐being, indicated by WHO‐5 scores < 50%. This is in‐line with the literature. Han et al. (2019) reported significantly lower serum irisin levels among patients with coronary heart disease (CHD) and add‐on depression when compared to CHD patients without depression or healthy controls. Furthermore, COPD patients with disturbed mood presented with lower circulating irisin in a study by Papp et al. (2017). Moreover, it was described that irisin levels predicted the incidence of post‐stroke depression assessed six months after study inclusion (Tu et al., 2018). In contrast, a study enrolling 98 obese women could not find any differences in irisin levels between patients with low and high depressiveness (Hofmann et al., 2016). However, those patients were morbidly obese (mean BMI = 49.2 kg/m^2^) and suffered various comorbidities.

The found connection between irisin and suspicious BDI levels was accompanied by an association between baseline irisin and (low‐grade) white matter lesions (*p* <.001). According to the vascular depression hypothesis, the accumulation of gliosis could be a key driver of late‐life depression (Herrmann et al., 2008; Krishnan et al., 2004). One of the potential mechanisms, by which irisin could be linked to white matter lesions, might be insulin resistance, which is associated with both increased with matter hyperintensities (Schur et al., 2015) and decreased irisin levels (Perakakis et al., 2017). However, this hypothesis could not be confirmed by our data, as BMI and HbA_1c_‐levels did not significantly moderate the association between the MRI findings and rank‐scaled, z‐standardized irisin, neither were they correlated with z‐standardized irisin levels in either of the groups (*p* >.05). Moreover, it needs to be further investigated why irisin was not linearly associated with white matter lesions, since only isolated lesions predicted decreased irisin levels, but not pronounced gliosis.

However, when predicting suspicious BDI levels by circulating levels of myokines/muscle‐activity‐related factors and group assignment (athletes/controls), the latter presented, indeed, as the strongest and solely significant predictor. Nevertheless, the model including the myokines significantly outperformed a model containing only the group status (*p* = .033). It is meanwhile well established that an individuals’ lifestyle must be taken into account when interpreting biomarkers. Physical activity, for example, affects various biomarkers and laboratory results, inter alia, by changes in blood volume, altered basal metabolism, and increased cellular turnover (Haslacher et al., 2015a, 2015b, 2017; Sanchis‐Gomar & Lippi, 2014). Myokines seem to follow this line, as their potential as biomarkers might be affected by physical activity.

### 4.2 KYN and IL‐6 are associated with depressive symptoms and reduced quality of life among controls

The same holds for kynurenine and IL‐6, which were significantly associated with several dimensions of the SF‐36 in controls, but not in athletes. Il‐6 correlated further with the WHO Quality of Life score (*ρ* = −0.287, *p* = .030), as well as with the Geriatric Depression Scale (*ρ* = 0.297, *p* = .025). The connection between IL‐6 and depression is well established (Goldsmith et al., 2016; Jin et al., 2020). Regarding the change in plasma KYN levels in depression, the literature is ambiguous. Whereas some report lower KYN concentrations (Colle et al., 2020; Pompili et al., 2019), others found no association between KYN levels and major depressive disorder (Bradley et al., 2015). In a recent meta‐analysis, however, it was shown that therapeutic immune activation by IFNα, which often induces depressive symptoms, for example, in patients with chronic Hepatitis C, was accompanied by a rise in peripheral KYN levels, suggesting a connection between KYN and the inflammatory pathogenesis of depression (Charlotte Hunt et al., 2020).

Among athletes, however, this association between circulating KYN‐ or IL‐6 levels and mood states was disturbed. A possible association between myokines and quality of life/depression could be masked among athletes by the comparably lower interindividual variability in the respective scores, which may have affected the correlation analyses. Moreover, physical activity may induce downstream‐reactions that have not been monitored in this study. Su et al. showed that KYN injections induced depression‐like behavior in non‐exercising, but not in exercising mice, which presented with overexpression of kynurenine aminotransferase III, which enhances KYN metabolism (Su et al., 2020). However, these findings imply that circulating myokines might have a limited predictive value regarding depressive symptoms and impaired quality of life among athletes, except for irisin levels.

### 4.3 CTSB is higher in athletes

We could show that CTSB concentrations were significantly higher in athletes than in controls (*U* = 962.5, *p* <.05). This molecule is a lysosomal cysteine protease that plays a variety of different roles, for instance, in tumor growth (Aggarwal & Sloane, 2014) and cell death (de Castro et al., 2016), whereby only some of them are considered beneficial. Above that, it has been demonstrated that CTSB is expressed and secreted by murine skeletal muscle in response to activity. This increase in CTSB levels led to an overexpression of the neuronal growth factor BDNF and doublecortin within the murine hippocampus, as well as to an improved outcome in the water maze test, suggesting a neuroprotective role for CTSB (Moon et al., 2016). In contrast to our findings, De la Rosa et al. (2019) reported a decrease in both CTSB and BDNF levels in amateur athletes in a dose‐dependent manner concerning the weekly training intensity. However, De la Rosa et al. (2019) recruited a heterogeneous sample of athletes in terms of age and sport disciplines, including veteran amateur rugby players and young individuals practicing tennis and taekwondo. The control group consisted of people who reported exercising less than 150 min per week. It has been shown before that IL‐6 facilitates CTSB expression in monocytes in tumor tissue, suggesting a potential connection between physical activity‐induced IL‐6 levels and CTSB. In fact, due to its short half‐life, IL‐6 may have dropped below the detection limit within a short period of time after a training session, whereas CTSB would still be detectable due to its markedly higher biological half‐life of ~14 hr (Katunuma, 2010). Except for CTSB, we found no other myokine/muscle activity‐related factor to be different between athletes and controls. As stated above, it must be kept in mind that those factors are not solely produced by the contracting muscle, but also by other tissues. Therefore, the circulating amount might not be fully attributable to physical activity. This is especially true for IL‐6, for which adipose tissue is a major source, explaining the association between IL‐6 and BMI/reduced physical performance among controls.

### 4.4 Myokine levels increase with age in controls

KYN, CTSB, irisin, and IL‐6 significantly increased with age among controls nearly to the same extend (ρ ~ 0.35), but not in athletes. This is in line with the literature, describing increasing levels of both plasma and cerebrospinal fluid irisin (Ruan et al., 2019), of IL‐6 (Ferrucci et al., 2005; Wei et al., 1992), KYN (de Bie et al., 2016; Refaey et al., 2017), and CTSB (Wyczałkowska‐Tomasik & Pączek, 2012). It is most likely the pro‐inflammatory shift in the metabolism of older adults, mainly due to increased oxidative stress that shifts the balance between neurotoxin and neuroprotective mediators in favor of the neurotoxic pathway (de Bie et al., 2016; Maggio et al., 2006). Moreover, in controls, higher IL‐6 levels were associated with impaired physical performance (*ρ* = −0.432, *p* = .008) and BMI (*ρ* = 0.435, *p* = .001), reemphasizing the connection between inflammation and physical capacities (Marsland et al., 2008). Especially the connection between IL‐6 and BMI is well established, as adipose tissue is considered one of the main sources of circulating IL‐6 (Carey et al., 2004). In athletes, an inverse correlation between the neurotoxin KYN and the neurotrophin BDNF emerged, however, without statistical significance (*ρ* = −0.254, *p* = .062). BDNF, again, showed a trend for being positively associated with training intensity [hr/week] (*ρ* = 0.263, *p* = .053).

Limitations were the sample size, which was too low to yield statistically significant results regarding the association between KYN, BDNF, and training intensity among athletes. Moreover, the necessity to transform CTSB and irisin levels due to considerable inter‐assay‐variability might decrease statistical power. Above that, the share of female participants was too low to be able to make statements with regard to sex. Finally, it might be considered a limitation that the included individuals do not represent a random sample of older marathoners, which could impair generalizability.

In conclusion, circulating myokines/muscle activity‐related factors like KYN and irisin and the multifunctional cytokine/myokine IL‐6 are associated with depressive symptoms among older adults, as data from our control cohort suggest. However, several of these associations appear to be diminished among athletes. It can only be speculated what might be the reason for this, however, it could be due to a shift of the balances in favor of anti‐inflammatory mediators, a more significant influence of other factors, as f.e. age, or small effect size for any of the parameters which might be masked by the small inter‐assay variability in the athletes’ quality of life scores. Hence, circulating myokine levels might be promising candidates to quantify the inflammatory component of depressive symptoms, but with limited applicability among athletes.

## AUTHOR CONTRIBUTIONS

**Patrick Mucher:** Conceptualization; Formal analysis; Investigation; Writing‐original draft. **Delgerdalai Batmyagmar:** Investigation; Writing‐review & editing. **Thomas Perkmann:** Investigation; Writing‐review & editing. **Manuela Repl:** Investigation; Writing‐review & editing. **Astrid Radakovics:** Investigation; Writing‐review & editing. **Elisabeth Ponocny‐Seliger:** Investigation; Methodology; Writing‐review & editing. **Ina Lukas:** Investigation; Methodology; Writing‐review & editing. **Monika Fritzer‐Szekeres:** Investigation; Resources; Writing‐review & editing. **Johann Lehrner:** Conceptualization; Investigation; Writing‐review & editing. **Thomas Knogler:** Methodology; Writing‐review & editing. **Dimiter Tscholakoff:** Investigation; Writing‐review & editing. **Martina Fondi:** Conceptualization; Writing‐review & editing. **Oswald Wagner:** Conceptualization; Resources; Supervision; Writing‐review & editing. **Robert Winker:** Conceptualization; Investigation; Methodology; Supervision; Writing‐review & editing. **Helmuth Haslacher:** Conceptualization; Data curation; Formal analysis; Investigation; Methodology; Project administration; Resources; Supervision; Visualization; Writing‐original draft.

## ETHICS STATEMENT

The initial trial (EK 401/2005, ClinicalTrials.gov NCT01045031), which included obtaining written informed consent and the present study (EK 2149/2019), were reviewed and approved by the ethics committee of the Medical University of Vienna. The approved research protocol (in German language) was uploaded to https://www.researchgate.net/publication/344237384_Myokinkonzentration_bei_alteren_MarathonatheltInnen_Expose_zur_Masterarbeit?_sg%5B0%5D=BT5wlyYyhyMH9OVTibJRQ7z‐vnB9mONK66OHDFrPP6wx9Qglh0CPqi_7f0oTnSqin_ipcMMwALX8fU‐Vtq2WQ7p4lSkWb9bikNLbI_EV.adKrLPEHoqyoqUA7MI7V17wFhtS8lW3JxPU2UluSZy9e6cqii4‐eRpJGjJK2xQOEXbkJWQwKA59HojwtKmOmwg.
